# Establishment and Cross-Protection Efficacy of a Recombinant Avian Gammacoronavirus Infectious Bronchitis Virus Harboring a Chimeric S1 Subunit

**DOI:** 10.3389/fmicb.2022.897560

**Published:** 2022-07-22

**Authors:** Xiong Ting, Chengwei Xiang, Ding Xiang Liu, Ruiai Chen

**Affiliations:** ^1^College of Veterinary Medicine, South China Agricultural University, Guangzhou, China; ^2^Zhaoqing Branch of Guangdong Laboratory of Lingnan Modern Agricultural Science and Technology, Zhaoqing, China; ^3^Integrative Microbiology Research Centre, South China Agricultural University, Guangzhou, China

**Keywords:** IBV, reverse genetics, cell adaptability, growth characteristics, immunization, broad-spectrum protection

## Abstract

Infectious bronchitis virus (IBV) is a gammacoronavirus that causes a highly contagious disease in chickens and seriously endangers the poultry industry. A diversity of serotypes and genotypes of IBV have been identified worldwide, and the currently available vaccines do not cross-protect. In the present study, an efficient reverse genetics technology based on Beaudette-p65 has been used to construct a recombinant IBV, rIBV-Beaudette-KC(S1), by replacing the nucleotides 21,704–22,411 with the corresponding sequence from an isolate of QX-like genotype KC strain. Continuous passage of this recombinant virus in chicken embryos resulted in the accumulation of two point mutations (G21556C and C22077T) in the S1 region. Further studies showed that the T248S (G21556C) substitution may be essential for the adaptation of the recombinant virus to cell culture. Immunization of chicks with the recombinant IBV elicited strong antibody responses and showed high cross-protection against challenges with virulent M41 and a QX-like genotype IBV. This study reveals the potential of developing rIBV-Beau-KC(S1) as a cell-based vaccine with a broad protective immunity against two different genotypes of IBV.

## Introduction

Coronaviruses are important pathogens of humans and animals and cause mainly enteric or respiratory diseases with clinical manifestations ranging from mild, self-limiting to severe and even life-threatening symptoms (Fung and Liu, 2019, 2021). Three newly emerging coronaviruses, severe acute respiratory syndrome coronavirus (SARS-CoV) in 2002–2003, Middle East respiratory syndrome coronavirus (MERS-CoV) in 2012, and most recently, SARS-CoV-2, are all thought to originate from bat coronaviruses, demonstrating the zoonotic potential of coronaviruses and their devastating impacts (de Wilde et al., 2018; Haake et al., 2020; Kandeel et al., 2020; Fung and Liu, 2021). Coronaviruses are enveloped single-stranded positive-stranded RNA viruses (+ss RNA), belong to the order Nidovirales (Wang et al., 2020), and are classified into four genera: alpha-, beta-, gamma-, and deltacoronavirus. Infectious bronchitis virus (IBV) is the first known member of the gammacoronavirus genus and was first discovered in North Dakota, USA, in 1931 (Liu et al., 2019). IBV mainly infects chickens and exhibits a wide range of tissue tropisms, causing damage to the respiratory, genitourinary, and digestive systems (Liu et al., 2019). Chickens aged from 1 to 4 weeks are most susceptible to the virus, with a mortality rate as high as 20% in severe cases (Ignjatović and Sapats, 2000; Hoerr, 2021). Besides, the disease appears to have a certain seasonal pattern, with a high incidence in winter and spring.

Due to the presence of a large host population and internal genetic diversity, IBV evolves rapidly. In addition, a high-frequency error rate of the IBV genome transcription and the frequent occurrence of recombination events lead to the emergence of multiple IBV variants (Jackwood, 2012; Legnardi et al., 2020), and new dominant strains are produced by selection and recombination from minor variants (Fang et al., 2005). Currently, nine genotypes of IBV, namely, QX (also known as LX4 type), Mass, 4/91, TW, CK/CH/LSC/99 I, J2, JP, Italy02, and Tc,07-2, have been classified (Qiu et al., 2019). Although with certain regional variations, the QX genotype is currently the main dominant strain that endangers the development of the poultry industry in China (Feng et al., 2015; Yan et al., 2019; Ren et al., 2020).

Infectious bronchitis virus has a limited tissue tropism and poor adaptability to passage in cell lines (Cunningham et al., 1972; Zhou et al., 2016; Bickerton et al., 2018b), which hampers the development and production processes of IBV vaccines. The existing commercial IBV vaccines are all traditional vaccines made in chicken embryos, using backward technologies with high production costs and a high risk of exogenous virus contamination. It is, therefore, imminent to develop a new generation of IBV vaccines with new technologies, such as reverse genetics. In this study, we report the construction of a recombinant IBV, rIBV-Beau-KC(S1), containing a chimeric S1 region by replacing nucleotides 21,704–22,411 in Beaudette-p65 with the corresponding sequence from an isolate of QX-like genotype KC strain. The rescued rIBV-Beau-KC(S1) exhibits both Vero cell adaptability and better cross-protection against the challenges with QX-like genotype strain CK/CH/JS/TAHY and Mass genotype strain M41. Besides, a G-C mutation at nucleotide 21,556 (G21556C, S248T) in the S1 region was confirmed to be essential for the cultivability of rIBV-Beau-KC(S1) in Vero cells. This study provides an option for the development of a novel IBV vaccine candidate with broad-spectrum protection.

## Materials and Methods

### Cell, SPF Chicken Embryo, and Virus

Vero, H1299, and HeLa cells were cultured at 37°C in Dulbecco's modified Eagle's medium (DMEM) supplemented with 10% fetal bovine serum (FBS) in the presence of penicillin (100 units/mL) and streptomycin (100 g/ mL) in a 5 % CO_2_ environment. SPF chicken embryos were purchased from Xinxing Dahuanong Poultry Egg Co., Ltd. (Guangdong, China). Vero cell-adapted IBV Beaudette strain (Beaudette-p65), obtained by adapting the egg-adapted Beaudette strain (ATCC VR-22) to Vero cells as previously described (Lim and Liu, 1998; Ng and Liu, 1998; Fang et al., 2005), was propagated in Vero cells in FBS-free DMEM. The CK/CH/JS/TAHY and CK/CH/GD/KPLH-CZQ 2018 strains (abbreviated as KC) were isolated from local chicken farms, the M41 strain was purchased from the Culture Collection Center of China Veterinary Drug Supervision Institute, and rIBV-siBeau was a double-silent mutant strain (A21963G and G22170C) of Beaudette-p65, and the strain was rescued in our laboratory.

### Animals and Ethics Statement

One-day-old SPF chickens were purchased from Xinxing Dahuanong Poultry Egg Co., Ltd. (Guangdong, China) for the immune efficiency assay. The study did not involve endangered or protected species and was approved by the Animal Experiments Committee of Zhaoqing Dahuanong Biopharmaceutical Co., Ltd.

### Construction of Full-Length rIBV-Beau-KC(S1) Containing the T7 Promoter

**Figure 1:**
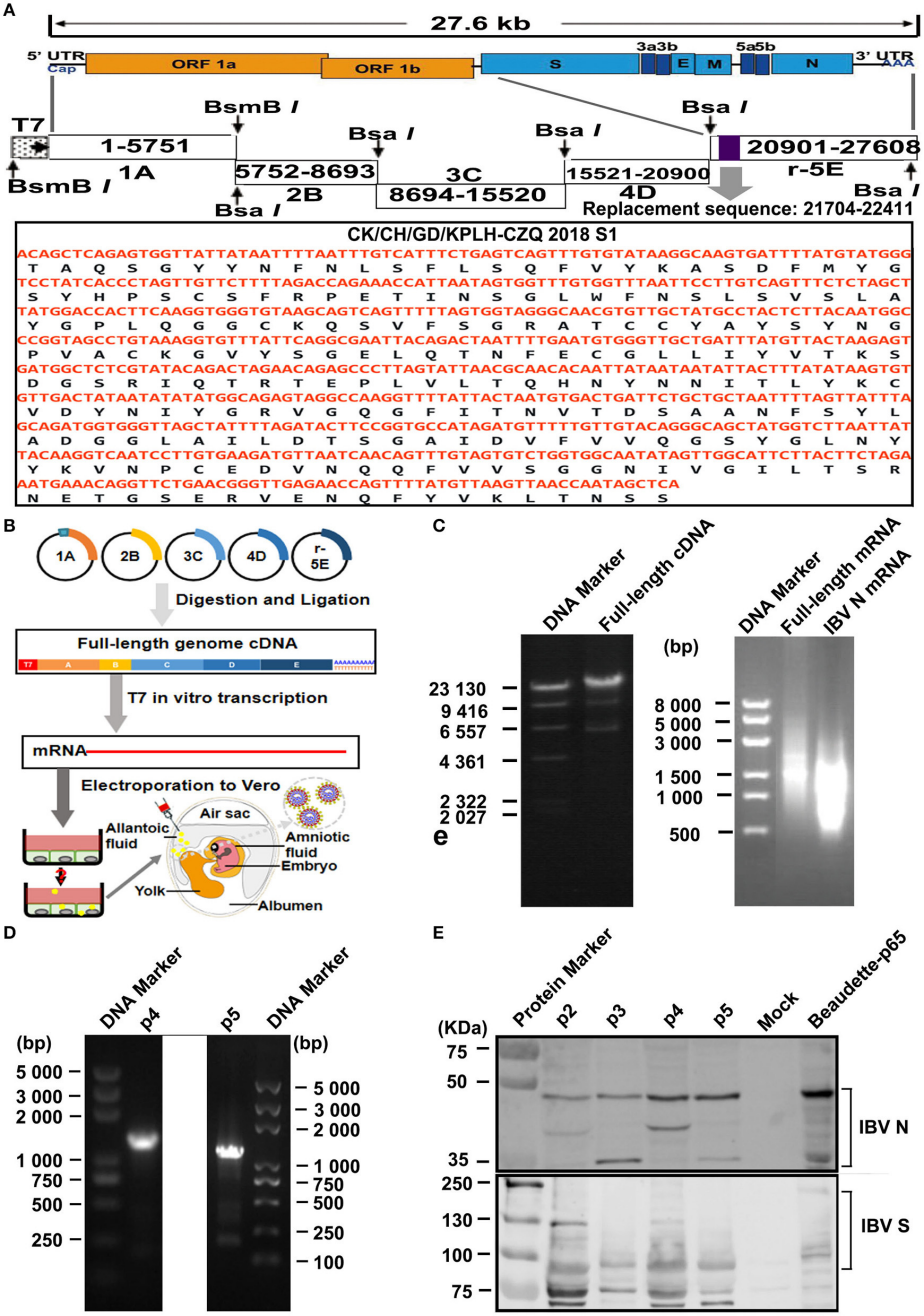
Construction and rescue of rIBV-Beau-KC(S1). **(A)** Diagram showing the genome organization of rIBV-Beau-KC(S1). The regions coding for the replicase polyproteins; the structural proteins S, E, M, and N; the accessory proteins 3a, 3b, 5a, and 5b; and the 5′- and 3′-UTR are shown. Also shown are the regions of the five RT-PCR fragments, the T7 promoter at the 5′-end of fragment A, the 30 As at the 3′ end of fragment E, and the replacement region of S1 at nucleotides 21,704–22,411. **(B)** The reverse genetics approach for the rescue of rIBV-Beau-KC(S1). **(C)** Assembly of the full-length cDNA clone and *in vitro* transcription of the full-length and IBV N transcripts. Equal amounts of the five purified fragments were ligated using T4 DNA ligase and analyzed on a 0.4% agarose gel. The *in vitro* assembled full-length and PCR-amplified IBV N DNA fragments were used as templates for generating the full-length and IBV N transcripts, which were analyzed on a 0.8% agarose gel. **(D)** RT-PCR identification of p4 and p5 using specific primers d-rIBV02F and d-rIBV02R. The total RNA was extracted from allantoic fluids of p4 and p5 and reverse transcribed into cDNA for RT-PCR identification. RT-PCR products were analyzed on a 0.8% agarose gel. **(E)** Western blot identification of p2-p5 using rabbit polyclonal antibody anti-IBV S and mouse monoclonal antibody anti-IBV N.

According to the manufacturer's protocol, total RNA was extracted from 200 μL of allantoic fluid of IBV KC strain using the Viral RNA Isolation Kit (Corning life sciences, CO., LTD, Wujiang, China), and was reverse transcribed into cDNA using FastKing RT SuperMix kit (TIANGEN Biotech CO., LTD, Beijing, China). The S1 region of the KC strain was amplified with forward primer PR-S1F and reverse primer PR-S1R (Table 1), and cloned into the pMD-19T vector for the construction of full-length cDNA of rIBV-Beau-KC(S1). The full-length cDNA was divided into five fragments and cloned into pKT-0, pGEM-T easy, pCR-TOPO-XL, pGEM-T easy, and pBR322(delete TcR+) vectors, respectively, generating plasmids pIBVcDNA-1A (1-5751), pIBVcDNA-2B (5752-8693), pIBVcDNA-3C (8694-15520), pIBVcDNA-4D (15521-20900), and pIBVcDNA-r5E (20901-27608). Plasmids pIBVcDNA-1A, pIBVcDNA-2B, pIBVcDNA-3C, and pIBVcDNA-4D were constructed as previously reported (Fang et al., 2007). Plasmid pIBVcDNA-r5E was constructed by replacing the nucleotides 21,704–22,411 of Beaudette-p65 with the corresponding sequence from an isolate of QX-like genotype KC strain (Figure 1A) by overlapping PCR and cloning the PCR fragment into pBR322 (delete TcR+) vector by homologous recombination. The related primers are listed in Table 1. The five fragments were prepared by digestion of the corresponding plasmids with Type IIS restriction enzyme Esp3I or BsaI (New England Biolabs (Beijing) LTD, Beijing, China) and gel extraction (QIAGENE, Germany). The full-length cDNA clone was seamlessly assembled from the purified fragments using T4 DNA ligase (New England Biolabs (Beijing) LTD. Beijing, China) and incubating overnight at 4°C.

**Table 1:** Primers for construction of pIBVcDNA-r5E plasmid and RT-qPCR.

**Primers**	**Sequences**	**Note**
PR-S1F	5′-AAGACTGAACAAAAGACCGACT-3	Amplification of full-length S1 of KC
PR-S1R	5′-CAAAACCTGCCATAACTAACATA-3′	
pBR322(delTcR+) F	5′-ACCCTAAGGGCGAATTCACCTCGCTAACGGATTCAC-3′	Construction of pIBVcDNA-r5E
pBR322(delTcR+) R	5′-CTATGGTCTCATTAGAGCCTCGTGATACGCCTAT-3′	
pre-5E F	5′-CTAATGAGACCATAGATGTTACATCTGCAGG-3′	
pre-5E R	5′-AACCACTCTGAGCTGTTTTTGTTTGGTAAGTTTGAATATTCTGAACACC-3′	
KC(S1) F	5′-ACAGCTCAGAGTGGTTATTATAATTTTAATTTGTC-3′	
KC(S1) R	5′-GAACGTCTAAAACGACGTGAGCTATTGGTTAACTTAACATAAAACTGG-3′	
fol-5E F	5′-CGTCGTTTTAGACGTTCTATTACTGAAAATG-3′	
fol-5E R	5′-GAATTCGCCCTTAGGGTCTCTT-3′	
d-rIBV02F	5′-TGGCCAGCTTTTCTATAATTTAAC-3′	Identification of rIBV-Beau-KC(S1)
d-rIBV02R	5′-GAGCACATTTTCAGTAACATT-3′	
IBVF	5′-CTATCGCCAGGGAAATGTC-3′	RT-qPCR for growth curve
IBVR	5′-GCGTCCTAGTGCTGTACCC-3′	
Probe	5′-FAM-CYTGGAAACGAACGGTAGACCCT-TAMRA-3′	

Coronavirus N gene transcript plays an accessory role to promote the recovery of the rescued virus (Casais et al., 2001; Youn et al., 2005). IBV N cDNA was obtained from pKTO-IBV N (Fang et al., 2007) containing the IBV N gene and the 3-UTR region by amplifying with forward primer PR-S1F and reverse prime PR-S1R (Table 1), and the PCR product was purified by using Omega cycle pure kit (Omega Bio-tek, Inc. Gerorgia, USA).

### *In vitro* Transcription and Electroporation

The full-length cDNA and N transcripts were generated *in vitro* using the mMessage mMachine T7 kit (Thermo Fisher Scientific, Inc. Massachusetts, USA) according to the manufacturer's instructions with certain modifications. Briefly, 10 μL of the transcription reaction mixture with 2 μL of template, 5 μL of 2 × CAP/NTP, 1 μL of 10 × buffer, and 1 μL of T7 RNApol enzyme was incubated at 37°C for 30 min, and then 1 μL of GTP was added and further incubated at 37°C for 150 min. The transcripts were extracted with phenol/chloroform.

The electroporation procedure was performed as previously reported with certain modifications (Yamada et al., 2009). Briefly, Vero cells in a 100 mm dish were grown to 90% confluence, trypsinized, washed twice with cold PBS, and resuspended in 900 μL of cold PBS. The full-length and N transcripts were introduced into Vero cells (300 μL) using the Gene Pulser X-cell electroporation system (Bio-Rad) at 110 V for 25 ms. The transfected Vero cells were cultured overnight in 1% FBS-containing DMEM in a six-well plate, the medium was then changed to FBS-free DMEM, and the cells were further incubated for 96 h and monitored for the appearance of CPE.

### Generating and Identifying the Rescued Virus rIBV-Beau-KC(S1)

The electroporated Vero cells were harvested and lysed by three freeze–thaw cycles at 96 h post-electroporation. The frozen/thawed stocks were subjected to consecutive passage in 9-day-old SPF chicken embryos (Yang et al., 2016). Total RNA was extracted from the allantoic fluid of p4 and p5 (Takara Bio Inc., Beijing, China) and reverse transcribed into cDNA (TIANGEN Biotech CO., LTD., Beijing, China) according to the manufacturer's instructions. Successful rescue of the recombinant virus was confirmed by PCR with forward primer d-rIBV02F and reverse primer d-rIBV02R (Table 1) and sequencing. The allantoic fluid of p2~p5 was further analyzed by Western blot analysis as described previously (Yuan et al., 2020; Zhu et al., 2021).

### The Cultivability of the Rescued Virus rIBV-Beau-KC(S1) in Vero Cells

Continuous passage of p4 and p10 was carried out in Vero cells to determine the cultivability of the rescued virus rIBV-Beau-KC(S1) in culture cells. In brief, 1 mL of allantoic fluid was inoculated into Vero cells in T25 plates with 9 mL of FBS-free DMEM. Vero cells were harvested and lysed by three freeze–thaw cycles at 72 h post-inoculation. Subsequently, 1 mL of virus stock was again inoculated into Vero cells in T25 plates with 9 mL of FBS-free DMEM for 72 h and repeated up to nine passages. CPE was observed in each passage. Besides, RT-PCR, Western blot, and immunofluorescence assay (IFA) were also performed to confirm the cultivability of rIBV-Beau-KC(S1).

Immunofluorescence assay was carried out as previously reported with certain modifications (Kohmer et al., 2020). Briefly, Vero cells were seeded on six-well plates and incubated at 37°C for 16 h. Cells were infected with IBV (MOI 0.2) for 2 h. The inoculum was then changed to a serum-free medium and continued to incubate at 37°C for 24 h. The supernatant was discarded, cells were washed two times with PBS for 5 min each and fixed with 50% acetone-formaldehyde mixture (v/v = 1/1) for 10 min, washed three times with PBS, and blocked with PBS containing 1% bovine serum albumin (BSA) plus Tween 20 [0.1% (vol/vol)] for 2 h at 37°C. Cells were incubated with the primary anti-IBV N antibody (QIANXUN Biological CO., LTD, Guangzhou, China) and with a secondary antibody [Goat Anti-Mouse IgG (H+L) Fluor488-conjugated antibody, Affinity Biosciences CO., LTD, US] for 2 h at room temperature, washed with PBST for three times, nuclear stained with 4′,6-diamidino-2-phenylindole (DAPI) for 8 min at room temperature, and washed with PBST. Images were obtained with a fluorescence microscope.

### Propagation and Infectivity of rIBV-Beau-KC(S1) in Vero, H1299, and HeLa Cells

To compare the replication of the rescued virus rIBV-Beau-KC(S1) and its parental strain Beaudette-p65 in cells (Vero, H1299, and HeLa), the two viruses infected the cells in six-well plates. At 12 or 24 h post-infection, IFA was used to detect if the cells were infected. Meanwhile, the infected cells were harvested at 0, 12, 24, 36, and 48 h post-infection (hpi) for real-time qPCR (RT-qPCR) assay. In brief, total RNA from the infected cells was extracted using the Viral RNA Isolation Kit (Corning life sciences, CO., LTD., Wujiang, China) according to the manufacturer's instructions. Reverse transcription was conducted at 42°C for 15 min using the FastKing-RT SuperMix Kit (TIANGEN Biotech CO., LTD, Beijing, China), and 9 μL of 10-fold diluted cDNA was analyzed by Taqman RT-qPCR to quantitatively detect the viral load. All qPCR reactions were carried out in triplicate, and the IBV copies were calculated based on a standard curve of a positive plasmid. Specific primers and probes of RT-qPCR are listed in Table 1 and purchased from Invitrogen (Thermo Fisher Scientific, USA), and AceQ Universal U+ Probe Master Mix V2 was purchased from Vazyme (Nanjing, China). The RT-qPCR was carried out in an ABI-QuantStudio 3 real-time PCR system.

To determine the EID_50_ and TCID_50_ values of rIBV-Beau-KC(S1), 10-fold serial dilutions of each viral stock were inoculated into 9-day-old SPF embryonated chicken embryos (ECEs) or Vero cells. For each dilution, 0.2 mL of the virus suspension was injected into each egg or cells in 96-well plates. Five eggs or plates were used for each dilution. The EID_50_ and TCID_50_ values were calculated by the method of Reed and Muench (1938).

### Immunization of Chickens and Virus Challenges

One-day-old SPF chickens (*n* = 15/group) were prime-immunized with a dose of 10^5^ EID_50_ rIBV-Beau-KC(S1) live vaccine by nasal-ocular route, and were boost-immunized intramuscularly with a dose of 10^5^ TCID_50_ rIBV-Beau-KC(S1)-inactivated vaccine at 14 days. The same dose of Beaudette-p65 and PBS was used as control.

At 35 days post-immunization (dpi), six chickens were randomly taken from each group to three isolators under negative pressure, and challenged with ~10^5.5^ EID_50_ of CK/CH/JS/TAHY strain by the nasal-ocular route, and the remaining chickens in each group were challenged with 2~3 drops (~10^7.45^ EID_50_) of IBV M41 strain at 84 dpi. The challenged chickens were then observed daily for clinical symptoms, such as tracheal rales, wheezing, nasal discharge, or death for 7 days. The surviving chickens in each group were euthanized at 7 days post-challenge, and necropsies were performed immediately after postmortem. Kidney tissues and tracheal tissues from the two challenged groups were collected and fixed in 10% neutral buffered formalin for further histopathological analysis, respectively.

### Antibody Detection and Cellular Immunoassay

Six serum samples were randomly taken from each group of chickens at 7, 14, 21, 28, 35, 42, 49, 56, 63, 70, 77, and 84 dpi. Total serum immunoglobulin G (IgG) specific for IBV was measured by indirect enzyme-linked immunosorbent assay (ELISA) (IDEXX, Westbrook, MA, USA) with certain modifications. Serum samples were 50-fold diluted and then detected by ELISA, and a positive sample was defined as absorbance of sample/positive control > 0.2. The positive serum samples that were 50-fold diluted were further 2-fold serially diluted, and endpoint titers were defined as the highest reciprocal serum dilution that yielded an absorbance >0.2.

The serum neutralization antibody against IBV was determined by the alpha method with certain modifications (Masoudi et al., 2020). Briefly, a 10-fold dilution of the KC (or M41) virus was prepared and mixed with a fixed dilution (1/5) of antiserum, and the doses of the minimum dilution of KC and M41 are 10^5.47^ EID_50_ and 10^6.01^ EID_50_, respectively. The mixture was incubated at room temperature for 1 h, and each mixture was inoculated into the group of five 9-day-old SPF ECEs. Endpoints were calculated by the Kärber or the Reed and Muench methods, and the results were expressed as a neutralization index (NI) that represented the log10 difference in the titers of the virus/negative serum mixtures and that of the virus/antiserum mixtures. NI ≥ 1.5 indicated a positive neutralization activity of the immunized serum.

Six anticoagulant peripheral blood samples were randomly taken from each group of chickens at 14 dpi and 28 dpi for flow cytometric analysis to detect the levels of CD4^+^, CD8^+^, Bu-1a^+^, and TCRγσ+. Six serum samples were randomly taken from each group of chickens at 7, 14, 21, and 28 days and pooled for further cytokine analysis by indirect ELISA (Cusabio Biotech, CO., LTD., Hubei, China) according to the manufacturer's instructions.

### Statistical Analysis

The data were analyzed using the GraphPad software package, and a two-way analysis of variance (ANOVA) was used to analyze significant differences between the indicated samples and the respective control samples. Significance levels are presented by the *P*-values (ns, non-significant; ^*^p < 0.05; ^**^p < 0.01; ^***^p < 0.0001).

## Results

### Rescue of the Recombinant Virus rIBV-Beau-KC(S1) Containing a Chimeric S1 Subunit

Using the reverse genetics approach described in the section “Materials and Methods,” the full-length cDNA clone of rIBV-Beau-KC(S1) was constructed by replacing the S1 region from nucleotides 21,704-22,411 of Beaudette-p65 with the corresponding region from an isolate of QX-like genotype KC strain (Figure 1A). The S1 region of QX-like genotype KC isolate was amplified and used to replace the corresponding region in fragment E, generating a new fragment E, r5E. The five fragments (1A to r5E) spanning the entire rIBV-Beau-KC(S1) genome were obtained by restriction digestion of the five fragments, which were then purified and ligated into the full-length cDNA (Figures 1B,C). Together with IBV N cDNA, the full-length cDNA was used for *in vitro* transcription to generate the full-length and N transcripts (Figure 1C).

Recovery of rIBV-Beau-KC(S1) from the cloned cDNA was performed according to a protocol that was successfully used for the recovery of a HiBiT-tagged recombinant IBV (Liang et al., 2020). Vero cells were electroporated with the *in vitro* transcribed full-length and N transcripts and cultured up to 96 h post-electroporation. As no obvious CPE was observed, cells were harvested and lysed by three freeze–thaw cycles. Total lysates were subsequently inoculated into the allantoic cavity of 9-day-old embryonated eggs for continuous blind passages to p10 (Figure 1B), and then analyzed by RT-PCR and Western blotting. Positive results were obtained by RT-PCR (Figure 1D) and Western blot analysis (Figure 1E) of the allantoic fluids collected from p2 to p5. The successful rescue of rIBV-Beau-KC(S1) was further confirmed by nucleotide sequencing.

### G21556C (S248T) Point Mutation in the S1 Region as a Determinant for the Cultivability of rIBV-Beau-KC(S1) in Vero Cells

**Figure 2:**
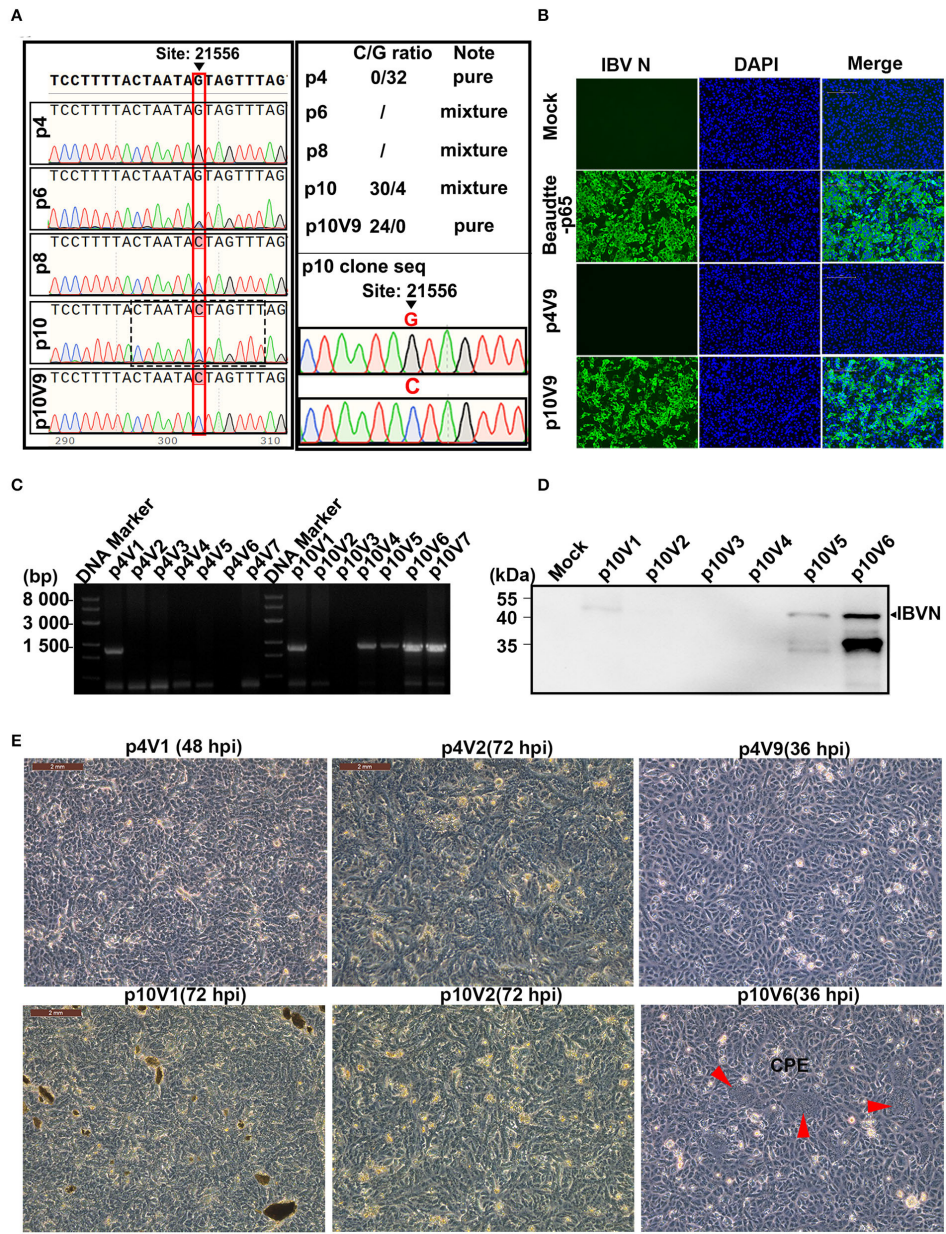
Effects of G21556C (S248T) point mutation in the S1 region on the cultivability of rIBV-Beau-KC(S1) in Vero cells. **(A)** Emergence and accumulation of G21556C mutation during the passage of rIBV-Beau-KC(S1) in chicken embryos. The regions covering the G21556C point mutation from p4, p6, p8, p10, and p10V9 were sequenced, showing double peaks of G and C in p6, p8, and p10. Mixed clones containing both G and C at the nucleotide position 21,556 were found in the 30 clones from p10, with the G/C ratio of 4:30, whereas the 32 clones from p4 contain only G and the 24 clones from p10V9 contain only C at the same position. **(B)** Immunofluorescent staining of Vero cells infected with rIBV-Beau-KC(S1). Vero cells were infected with rIBV-Beau-KC(S1)-p4V9 and rIBV-Beau-KC(S1)-p10V9 at MOI ~0.2, respectively. Vero cells were also mock-treated and infected with Beaudette-p65 as negative and positive controls. Cells were analyzed by IFA staining with an anti-IBV N monoclonal antibody and goat-mouse IgG-Fluor488 secondary antibody at 24 hpi. **(C)** RT-PCR analysis of Vero cells infected with rIBV-Beau-KC(S1). Vero cells were consecutively infected with rIBV-Beau-KC(S1)-p4 and rIBV-Beau-KC(S1)-p10, total RNAs were extracted, and the viral replication was checked by RT-PCR using specific primers d-rIBV02F. The PCR products were analyzed on a 0.8% agarose gel. **(D)** Western blot analysis of Vero cells infected with rIBV-Beau-KC(S1). Vero cells were infected with rIBV-Beau-KC(S1)-p10V1-V6, and harvested at 24 hpi. Total cell lysates were prepared and subjected to Western blot analysis with anti-IBV N mouse monoclonal antibody and goat-mouse IgG-HRP secondary antibody. **(E)** Formation of syncytia in Vero cells infected with rIBV-Beau-KC(S1). Continuous passages of rIBV-Beau-KC(S1)-p4 and p10 were carried out in Vero cells, and the formation of syncytium cells was observed in cells infected with rIBV-Beau-KC(S1)-p10V6 at 36 hpi.

Sequencing of the replacement region from p4, p6, p8, and p10 was then carried out, further proving that the recombinant virus was successfully rescued. However, a single C22077T point mutation was found in p4 and p6, and two point mutations (G21556C and C22077T) were found in p8 and p10, respectively. It was noted that double peaks of G and C were detected in the G21556C position in p6, p8, and p10, suggesting the presence of both quasispecies in these passages (Figure 2A).

As the initially rescued rIBV-Beau-KC(S1) appears to be unable to grow in culture cells, it would be interesting to see if this recombinant virus could be adapted to cell culture, and if so, the amino acid residue(s) responsible would be determined. For this purpose, the continuous passage of p4 and p10 in the Vero cells was carried out initially by inoculating 1 mL of the corresponding allantoic fluid into the Vero cells cultured in T25 plates with 9 mL of FBS-free DMEM for 72 h, and harvested as virus stocks by three freeze–thaw cycles. Subsequently, 1 mL each of these virus stocks was used to infect Vero cells in T25 plates, and the process was repeated up to nine passages. The initial virus stocks harvested from the infected Vero cells were labeled as rIBV-Beau-KC(S1)-p4V1 and rIBV-Beau-KC(S1)-p10V1, and the virus stocks harvested from the last passage were labeled as rIBV-Beau-KC(S1)-p4V9 and rIBV-Beau-KC(S1)-p10V9, and so on (Figures 2B–E).

At 36 h post-infection, a typical CPE of the Vero cell-adapted IBV, that is, the formation of giant syncytium cells, was observed in cells infected with rIBV-Beau-KC(S1)-p10V6, which carries G21556C and C22077T mutations, but no CPE was observed in the cells infected with rIBV-Beau-KC(S1)-p4V9 carrying the C22077T mutation only (Figure 2E). Both RT-PCR and Western blot analyses confirmed that rIBV-Beau-KC(S1)-p10V6 efficiently infected and replicated in Vero cells (Figures 2C,D). On the other hand, the passage of rIBV-Beau-KC(S1)-p4 in Vero cells showed a gradual loss of the viral infectivity, and the viral-specific band could not be detected after two passages (Figure 2C), indicating that the G21556C point mutation within the S1 region is a determinant for the cultivability of IBV in Vero cells.

The initial occurrence of the G21556C point mutation in rIBV-Beau-KC(S1) was investigated by cloning RT-PCR products covering the replacement region from rIBV-Beau-KC(S1)-p4, rIBV-Beau-KC(S1)-p10, and rIBV-Beau-KC(S1)-p10V9, respectively, to pMD-19T, and 24-32 single colonies from each plate were picked for sequencing. The results showed that all p4 clones had a G at position 21,556 (G21556), and all p10V9 clones contained a C at the same position (C21556). However, mixed clones were found in the 30 clones from p10, with a G/C ratio of 4:30 (Figure 2A). Immunofluorescent staining of infected cells using an anti-IBV N monoclonal antibody and goat-mouse IgG-Fluor488 secondary antibody at 24 hpi confirmed that rIBV-Beau-KC(S1)-p10V9 grew well in Vero cells and caused a typical CPE, whereas rIBV-Beau-KC(S1)-p4 could not grow in Vero cells (Figure 2B). These results demonstrate that the G21556C mutation emerged during the adaptation of rIBV-Beau-KC(S1) in chicken embryos and is essential for the cultivability of the virus in culture cells.

### The Growth Properties and Kinetics of rIBV-Beau-KC(S1) in Culture Cells and in Chicken Embryos

**Figure 3:**
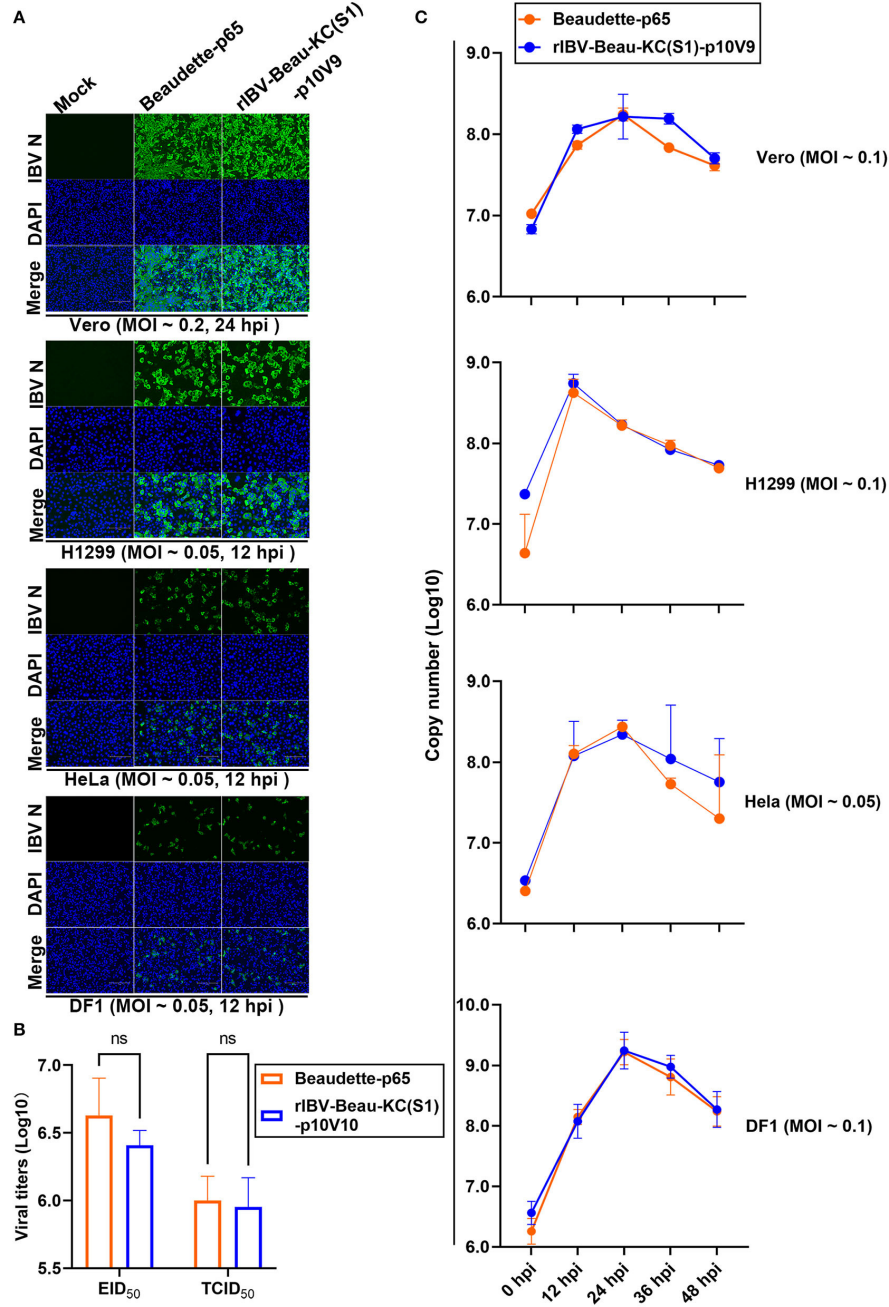
Replication of rIBV-Beau-KC(S1) in Vero, H1299, HeLa, and DF1 cells. **(A)** IFA of Vero, H1299, HeLa, and DF1 cells infected with rIBV-Beau-KC(S1). Cells were infected with rIBV-Beau-KC(S1)-p10V9 and Beaudette-p65 at an MOI ~0.2 or ~0.05, respectively. IFA was performed with anti-IBV N monoclonal antibody and goat-mouse IgG-Fluor488 secondary antibody. **(B)** Determination of the maximum EID_50_ and TCID_50_ values of rIBV-Beau-KC(S1). Ten-fold serial dilutions of each viral stock were inoculated into 9-day-old SPF ECEs or Vero cells. For each dilution, 0.2 mL of virus suspension was injected into each egg or added to cells in 96-well plates. Five eggs or plates were used for each dilution. The EID_50_ and TCID_50_ values were calculated by the method of Reed and Muench. Two-way analysis of variance (ANOVA) was used to analyze significant differences between the indicated samples and the respective control samples. Significance levels are presented by the P-value (ns, non-significant). **(C)** Growth kinetics of rIBV-Beau-KC(S1) in Vero, H1299, HeLa, and DF1 cells. Cells were infected with rIBV-Beau-KC(S1)-p10V9 and Beaudette-p65 at an indicated MOI, harvested at indicated time post-infection, and total RNAs were extracted. An equal volume of total RNA was reverse transcribed, and the levels of IBV genomic RNA were determined by RT-qPCR.

The growth properties of this recombinant virus in culture cells were first studied by infecting Vero, H1299, HeLa, and DF1 cells with Beaudette-p65 and rIBV-Beau-KC(S1)-p10V9, respectively. The infected cells were stained with anti-IBV N monoclonal antibody at 12 or 24 hpi, confirming that both Beaudette-p65 and rIBV-Beau-KC(S1)-p10V9 can efficiently replicate in all the four cell lines (Figure 3A).

The EID_50_ and TCID_50_ values of rIBV-Beau-KC(S1)-p10V9 in the chicken embryos were determined. As shown in Figure 3B, the maximum EID_50_ and TCID_50_ values of rIBV-Beau-KC(S1)-p10V9 reached 10^6.41^ and 10^5.95^, respectively, with no statistically significant difference from the maximum EID_50_ (10^6.63^) and TCID_50_ (10^5.99^) values of Beaudette-p65 (Figure 3B).

The growth curves of Beaudette-p65 and rIBV-Beau-KC(S1)-p10V9 in Vero, H1299, HeLa, and DF1 cells were then determined by infecting the four cell lines with Beaudette-p65 and rIBV-Beau-KC(S1)-p10V9, respectively, at an indicated MOI. The two viruses showed similar growth characteristics, reaching peaks at 24 hpi in Vero, HeLa, and DF1 cells, and at 12 hpi in H1299 cells (Figure 3C).

### Effects of G21556C Point Mutation on the Binding, Entry, and Infectivity of rIBV-Beau-KC(S1) in Culture Cells

**Figure 4:**
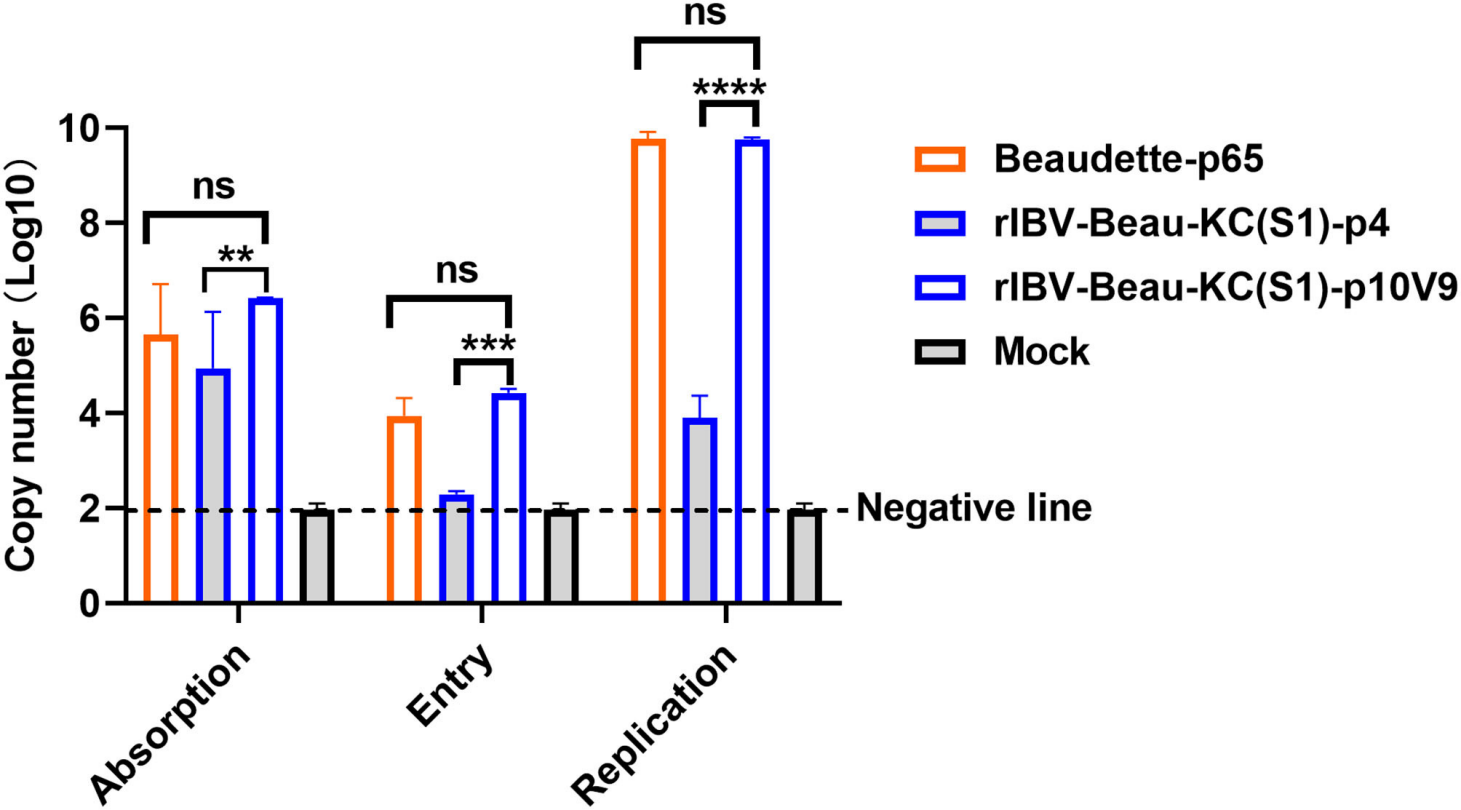
Effects of G21556C (S248T) point mutation on the absorption, entry, and replication of rIBV-Beau-KC(S1) in Vero cells. For determining the efficiency of viral absorption, entry, and replication, Vero cells were infected with rIBV-Beau-KC(S1)-p4, rIBV-Beau-KC(S1)-p10V9, and Beaudette-p65 at an MOI ~ 0.5, respectively. Cells were then treated and harvested essentially as described in the text, and total RNA was extracted. The levels of IBV genomic RNA were determined by RT-qPCR. Two-way analysis of variance (ANOVA) was used to analyze significant differences between the indicated samples and the respective control samples. Significance levels are presented by the *P*-value (ns, non-significant; **p < 0.01; ***p < 0.001; ****p < 0.0001).

The influence of G21556C point mutation on each step of the rIBV-Beau-KC(S1) replication cycle was then studied. The binding activity of the virus to Vero cells was assessed by adding Beaudette-p65, rIBV-Beau-KC(S1)-p4, and rIBV-Beau-KC(S1)-p10V9 to Vero cells and incubating at 4°C for 2 h. After removal of the unbound viruses by washing the cells with PBS for three times, the RNA copy numbers of the bound viruses were determined by RT-qPCR. As shown in Figure 4, significantly more absorption of rIBV-Beau-KC(S1)-p10V9 than rIBV-Beau-KC(S1)-p4 was found, suggesting that the G21556C point mutation did affect the binding of the virus to Vero cells.

To assess their entry efficiency, the unbound viruses were removed by digestion with 0.25% trypsin. The cells were then incubated at 37°C for 2 h after thorough washing, and the viral RNA copy numbers were determined. The results showed that the RNA copy number of rIBV-Beau-KC(S1)-p10V9 was similar to that of Beaudette-p65, but rIBV-Beau-KC(S1)-p4 can hardly be detected (Figure 4). Further incubation of the cells at 37°C for 24 h showed highly efficient replication of rIBV-Beau-KC(S1)-p10V9 and Beaudette-p65, but much reduced replication of rIBV-Beau-KC(S1)-p4 was observed (Figure 4). These results confirm that the G21556C point mutation has a profound effect on the binding and entry of rIBV-Beau-KC(S1) into the culture cells.

### Immunization of SPF Chickens With rIBV-Beau-KC(S1) Vaccine Candidate

**Figure 5:**
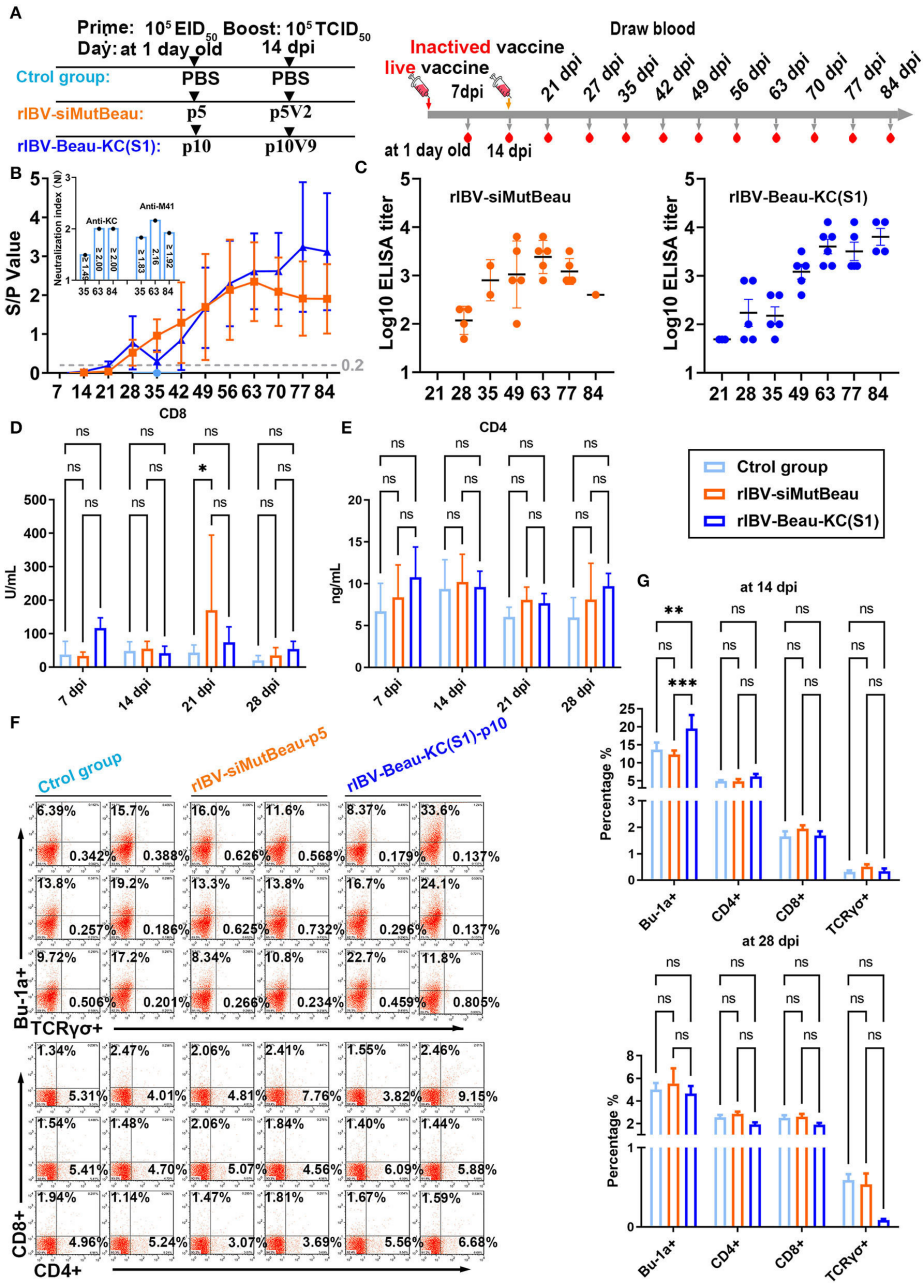
Humoral and cellular immune efficiencies in chickens immunized with rIBV-Beau-KC(S1). **(A)** Immunization and exsanguination protocol. Prime immunization with PBS, and 105EID50 rIBV-siMutBeau-p5- and 105EID50 rIBV-Beau-KC(S1)-p10-inactivated vaccines, respectively. Boost immunization with PBS, and 105 TCID50 rIBV-siMutBeau-p5V2- and 105 TCID50 rIBV-Beau-KC(S1)-p10V9-activated vaccines, respectively. Blood samples were collected weekly after immunization. **(B)** Vaccination-induced IBV-specific IgG in SPF chickens. One-day-old chicks were immunized with live or inactivated IBV vaccine with a booster 14 days later. Antibody response was monitored in the serum samples by commercial ELISA kit (IDEXX laboratories, USA) at weekly intervals till 84 dpi (*n* = 3 or 6/group). Serum neutralization indexes (NI) of rIBV-Beau-KC(S1)-immunized group against KC (QX-like genotype) and M41 were determined by alpha method with certain modifications. KC (10^6.47^ EID_50_/mL) and M41 (10^7.01^ EID_50_/mL) were 10-fold serially diluted, and the titers of each viral dilution were determined after mixing with a fixed dilution (1/5) of serum from the immunized chicks. NI represents the log10 difference between the titer of a viral dilution mixed with the immunized serum and the titer of the same viral dilution treated with the negative serum, and NI > 1.5 indicates a positive neutralization activity of the immunized serum. **(C)** Serum anti-IBV antibody titers were determined by ELISA (IDEXX laboratories, USA) at the indicated time points. The positive serum samples with 50-fold dilution were 2-fold serially diluted, and endpoint titers were defined as the highest reciprocal serum dilution that yielded an absorbance >0.2. **(D,E)** The levels of CD4 and CD8 molecules representing the activation of CD4+ and CD8+ cells in the serum samples were determined by ELISA (CUSABIO, China) at the indicated time points. Two-way analysis of variance (ANOVA) was used to analyze significant differences between the indicated samples and the respective control samples. Significance levels are presented by the *P*-value (ns, non-significant; *p < 0.05; **p < 0.01; ***p < 0.0001). **(F)** The levels of Bu-1a+, TCRγσ+, CD4+, and CD8+ in peripheral blood samples at 14 dpi were detected by flow cytometry. **(G)** Statistical analysis for flow cytometric results at 14 dpi and 28 dpi by GraphPad prism 9. Two-way analysis of variance (ANOVA) was used to analyze significant differences between the indicated samples and the respective control samples. Significance levels are presented by the *P*-value (ns, non-significant; *p < 0.05; **p < 0.01; ***p < 0.0001).

To evaluate the immunogenicity of rIBV-Beau-KC(S1), 1-day-old SPF chickens were immunized with rIBV-Beau-KC(S1) and rIBV-siMutBeau, respectively, and exsanguinated, according to the process listed in Figure 5A. The anti-IBV antibody response in chicks immunized with rIBV-Beau-KC(S1) and rIBV-siMutBeau began to turn positive at 21 and 28 dpi, respectively (Figure 5B). The average antibody titers in chicks immunized with rIBV-Beau-KC(S1) at 63, 77, and 84 days post-immunization were 1:5,333, 1:4,800, and 1:8,000, respectively, which were much higher than the corresponding titers of 1:3,039, 1:1,440, and 1:399 in the rIBV-siBeau group (Figure 5C). Quantification of the neutralizing antibody titers (NAb) to KC and M41, respectively, by alpha NAb test (VNT) showed the neutralization index (NI) ≥1.5 in chicks immunized with rIBV-Beau-KC(S1), demonstrating that immunization of chicks with this recombinant virus could elicit comparable levels of cross-neutralizing antibodies against both KC and M41, with slightly higher titers against M41 than KC (Figure 5B).

The determination of CD4+ and CD8+ cells by flow cytometric analysis and indirect ELISA showed that very similar levels of both cell types were stimulated in chicks immunized with rIBV-Beau-KC(S1), rIBV-siBeau vaccine, and control groups (Figures 5D–F). Consistent with the antibody titers elicited, the levels of Bu-1a+ (B-cell marker) induced in chicks immunized with rIBV-Beau-KC(S1) at 14 dpi were significantly higher than those in the rIBV-siMutBeau group (Figure 5G). Taken together, these results demonstrate that immunization of chicks with rIBV-Beau-KC(S1) mainly elicits humoral immunity.

### Cross-Protection Against Challenges With Virulent Strains CK/CH/JS/TAHY and M41 in Chickens Immunized With rIBV-Beau-KC(S1)

**Figure 6:**
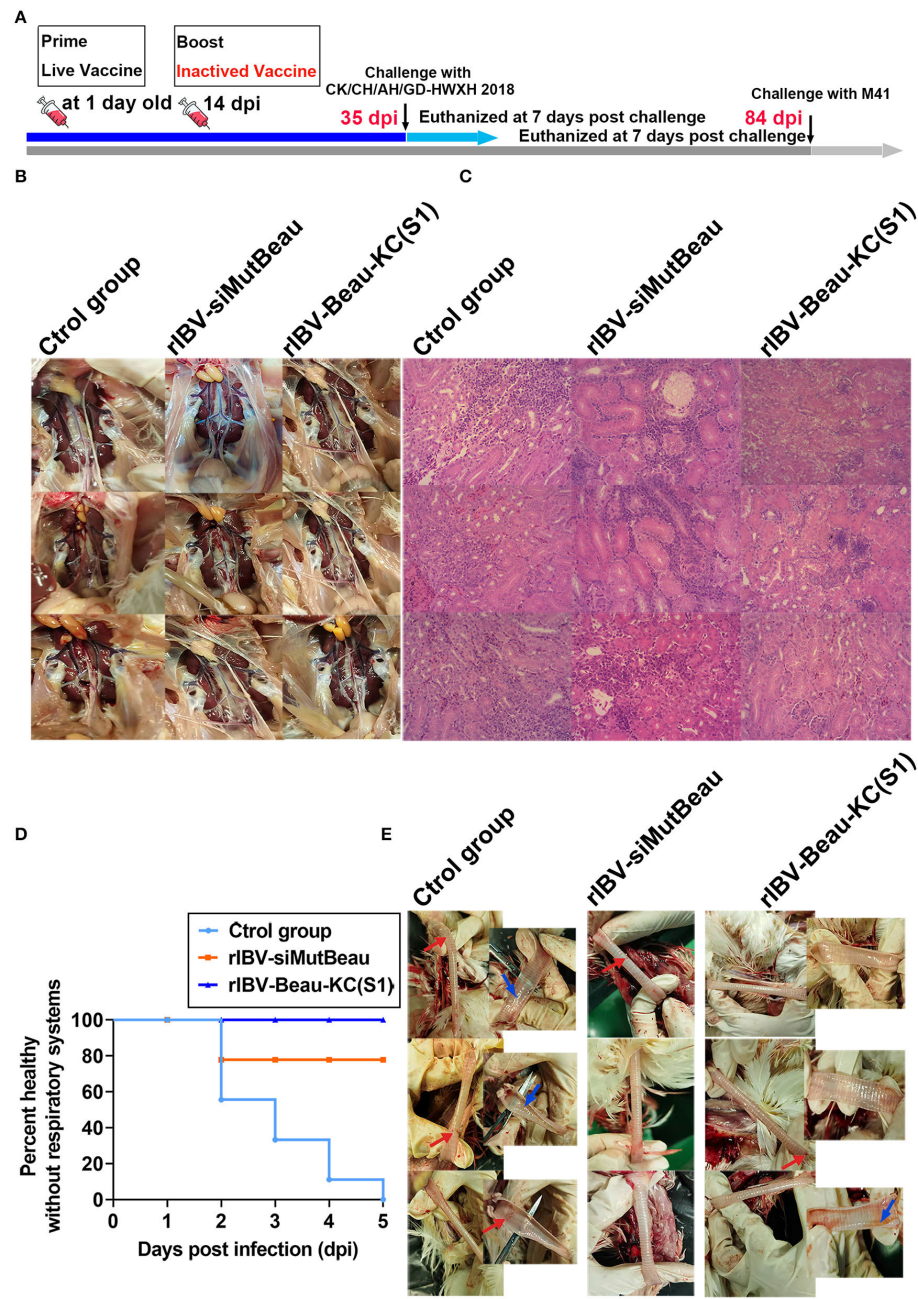
Cross-protection against CK/CH/JS/TAHY and M41. **(A)** Immunization and challenge protocol. One-day-old chicks (*n* = 15/group) were prime-immunized with a dose of 10^5^ EID_50_ rIBV-Beau-KC(S1) live vaccine by nasal-ocular route, and were boost-immunized intramuscularly with a dose of 10^5^ TCID_50_ rIBV-Beau-KC(S1)-inactivated vaccine at 14 days. The same doses of rIBV-siMutBeau and PBS were used as control, and CK/CH/JS/TAHY and M41 challenges were performed at 35 and 84 dpi, respectively. **(B,C)** At 7 days post-challenge with ~10^5.5^ EID_50_ of CK/CH/JS/TAHY, three chickens were randomly selected from each experimental group, an autopsy was done, and HE stained pathological tissue sections were observed. No obvious macroscopic lesions in the kidneys were observed. HE sections confirmed that the structure of the kidneys of all chickens in the rIBV-Beau-KC(S1)-immunized group was normal, except in one (33.33%) with slight congestion and hemorrhage. However, degeneration and necrosis of renal tubular epithelial cells, inflammatory cell proliferation, and infiltration were seen in kidneys (100%) obtained from all chickens in both control and rIBV-siMutBeau-immunized groups. **(D)** Within 5 days post-challenge with ~10^7.45^ EID_50_ of M41, the occurrence of tracheal rales in each experimental group was recorded and shown. Chicks in the rIBV-Beau-KC(S1)-immunized group had no clinical symptoms, but all chickens (100%) in the control group had obvious tracheal rales within 5 days post-challenge, and two chickens (22.22%) in the rIBV-siMutBeau-immunized group also had slight tracheal rales at 2 days post-challenge. **(E)** The necropsy results of the trachea of three randomly selected chickens at 7 days post-challenge with ~10^7.45^ EID_50_ of M41. Tracheas from all the three chicks (100%) in the control group had hemorrhage dots (red arrows) and two (66.67%) had obvious translucent mucus (blue arrows). One chick had hemorrhage dots (33.33%) in the rIBV-siMutBeau-immunized and rIBV-Beau-KC(S1)-immunized groups.

The protective efficacy of rIBV-Beau-KC(S1) against challenges with virulent strains of QX-like genotype (CK/CH/JS/TAHY) and Mass genotype (M41) was evaluated by infection of five immunized chickens in each group at 35 and 84 dpi, respectively (Figure 6A).

Within 7 dpi post-challenge with ~10^5.5^ EID_50_ of CK/CH/JS/TAHY, chickens in the rIBV-Beau-KC(S1)-immunized group showed no obvious clinical symptoms, whereas two (33.33%) in the control group and one (16.67%) in the rIBV-siMutBeau-immunized group showed mild symptoms, including poor spirit and fluffy feathers. At 7 days post-challenge, three chickens in each experimental group were randomly euthanized and necropsied, showing no obvious macroscopic lesions in kidneys (Figure 6B). Further evaluation of the kidney damage by routine HE sections confirmed that kidney structures of all chickens in the rIBV-Beau-KC(S1)-immunized group were normal, except in one (33.33%) with slight congestion and hemorrhage. However, degeneration and necrosis of renal tubular epithelial cells, inflammatory cell proliferation, and infiltration were seen in kidneys (100%) obtained from all chickens in both control and rIBV-siMutBeau-immunized groups (Figure 6C).

Within 5 days post-challenge with 2~3 drops (~10^7.45^ EID_50_) of M41, chickens in the rIBV-Beau-KC(S1)-immunized group had no clinical symptoms, but all chickens (100%) in the control group had obvious tracheal rales within 5 days post-challenge, and two chickens (22.22%) in the rIBV-siMutBeau-immunized group also had slight tracheal rales at 2 days post-challenge (Figure 6D). At 7 days post-challenge, three chickens in each group were randomly euthanized and necropsied. Tracheas from all three chickens (100%) in the control group had hemorrhage dots (red arrows), and two (66.67%) showed obvious translucent mucus (blue arrows), while trachea (33.33%) from one chicken in the rIBV-siMutBeau-immunized group had hemorrhage dots (Figure 6E). It was also noted that trachea from one chicken (33.33%) in the rIBV-Beau-KC(S1)-immunized group had yellow-red mucus, apparently a mixture of small amounts of blood and esophageal contents (Figure 6E), probably due to an injury to the trachea during euthanization.

## Discussion

Current prevention and control of IBV infection mainly relied on vaccine immunity (Sjaak et al., 2011; Jordan, 2017). Unfortunately, due to the presence of a large number of IBV variants worldwide (Lin and Chen, 2017; Domanska-Blicharz et al., 2020; Tegegne et al., 2020), most currently available vaccines have poor cross-protection. For example, the widely used vaccines of the Mass genotype have limited cross-protection against different genotypes, frequently resulting in vaccination inefficiency and failures (Jordan, 2017; Legnardi et al., 2020). These commercial vaccines are either live-attenuated or inactivated, both relying on the adaptation and propagation of isolates in chicken embryos with backward technologies. Furthermore, a majority of field isolates including QX-like genotype, the most relevant genotypes currently circulating worldwide, lacks cell adaptability (Laconi et al., 2020). Over the past decades, the development and application of novel vaccine technologies against IBV infection have been slow, and are hindered by the constraints of large-scale poultry production (Laconi et al., 2020). In this study, a reverse genetics technology was applied to successfully construct a recombinant virus rIBV-Beau-KC(S1) with both cell adaptability and better cross-protection against IBV infection.

Coronavirus spike (S) glycoprotein is a homo-trimer and the main immunogenic protein that induces neutralizing antibodies (Keep et al., 2020). It also plays an essential role in virulence *in vivo* and cellular tropism *in vitro* (Bickerton et al., 2018c). S protein is cleaved by host proteases to generate S1 and S2 subunits (Yamada and Liu, 2009; Bouwman et al., 2020a). As a determinant for cell tropism (Casais et al., 2003; Britton et al., 2005), the S1 subunit mediates the initial attachment of the virus, while the S2 subunit, containing ectodomain, a transmembrane domain, and endodomain, drives virus–cell fusion (Heald-Sargent and Gallagher, 2012; Wickramasinghe et al., 2014).

Similar to other coronaviruses, IBV generally exhibits restricted cell and tissue tropism, depending upon the S glycoprotein of individual strains (Sánchez et al., 1999; Tekes et al., 2010). Some IBV strains are able to replicate in primary chicken cells, such as chick kidney (CK) cells (Oade et al., 2019) and chick embryo fibroblasts (DF1) (Harrison et al., 2007; Huang et al., 2021). Interestingly, the Beaudette strain shows an extended host range with the ability to replicate in Vero and a number of human and other animal cell lines (Fang et al., 2005; Tay et al., 2012), as well as in baby hamster kidney (BHK-21) cell line after passaging in embryonated eggs (Otsuki et al., 1979; Fang et al., 2005). In this study, the rescued rIBV-Beau-KC(S1) strain with two point mutations apparently acquired an extended host range with the ability to grow in Vero, H1299, and HeLa cells, with similar growth characteristics as Beaudette-p65. As the cell-culturable rIBV-Beau-KC(S1)-p10V9 and Beaudette-p65 share the same G21556C (S248T) substitution in the S1 subunit, the T248 residue at this position may play a crucial role in determining the cell adaptability of IBV. The underlying mechanism is yet to be revealed, and the available evidence suggests that this substitution may enhance the binding and entry of the recombinant virus into the cells, consequently affecting the viral replication in culture cells.

The binding of S protein to a specific receptor(s) largely determines the host and tissue range of a coronavirus. Specific protein receptors for many alpha- and betacoronaviruses have been identified (Belouzard et al., 2012; Promkuntod et al., 2014). However, neither the exact receptor(s) for IBV (being a gammacoronavirus) nor the location of the receptor-binding domain (RBD) in IBV S protein has been unambiguously defined. Alpha-2,3-linked sialic acids have been implicated to be essential for the attachment and infection of IBV (Wickramasinghe et al., 2011; Promkuntod et al., 2014), and N-glycosylation of IBV S protein is a determinant for receptor-binding specificity (Parsons et al., 2019; Bouwman et al., 2020a,b). It is yet to be determined if the G21556C (S248T) substitution would change the receptor-binding affinity of the S protein by either directly enhancing the receptor binding or altering the overall 3-D structure of the protein. As the G21556C (S248T) substitution is outside the replacement region, this substitution would occur as a compensatory mutation to restore the binding affinity of the chimeric S1 subunit to the cellular receptor(s)/attachment factor(s) during the adaptation of this recombinant virus to chicken embryos and culture cells. Determination of the 3-D structure of this chimeric S protein would be of help to clarify this issue.

Recombinant IBVs expressing heterologous S proteins have been generated previously. Based on the Beaudette genome with only the S (or S1 region) gene derived from either H120, M41, 4/91, or QX, recombinant IBVs were constructed by chimerizing the S genes (or S1 region) and rescued (Bickerton et al., 2018a; Jiang et al., 2020). For example, rBeau-H120 (S1e) has the potential to be an alternative vaccine against IBV based on its excellent propagation property and immunogenicity (Wei et al., 2014); both BeauR and BeauR-M41(S) induced protection against challenges with M41 (Hodgson et al., 2004); BeauR-4/91(S) conferred protection against challenges with wild type 4/91 virus and a degree of heterologous protection against M41 (Armesto et al., 2011); both homologous and heterologous vaccination with BeauR-M41(S) and BeauR-4/91(S) reduced clinical signs and birds recovered more rapidly (Keep et al., 2020); a single vaccination of SPF chickens with rIBV expressing S1 of virulent strain M41 or QX, BeauR-M41(S1) and BeauR-QX(S1), provided incomplete protection against homologous challenge (Ellis et al., 2018). The construction of rIBV-Beau-KC(S1) and evaluation of its immunogenicity in this study adds a new example showing that a recombinant IBV carrying a chimeric S1 subunit may elicit a more potent and broadly protective immune response. The mechanisms underlying this better protection efficacy are yet to be elucidated. Immunization of chicks with rIBV-Beau-KC(S1) elicited a higher antibody response, which may render a better protection efficacy. Alternatively, this recombinant virus might be able to induce higher specific T-cell responses. Further studies are required to address this possibility.

In summary, an efficient reverse genetics system was used to construct and rescue rIBV-Beau-KC(S1) containing a chimeric S1. Continuous passage of rIBV-Beau-KC(S1) in chicken embryos resulted in the accumulation of two point mutations, in which the S248T (G21556C) substitution was confirmed to play a crucial role in determining the cell adaptability of IBV. Immunization of chicks with rIBV-Beau-KC(S1) elicited higher antibody responses than did rIBV-siMutBeau and showed better cross-protection against challenges with M41 and CK/CH/JS/TAHY (QX-like genotype), revealing the potential of developing rIBV-Beau-KC(S1) as a cell-based vaccine with broad-spectrum protection against two different genotypes of IBV.

## Data Availability Statement

The original contributions presented in the study are included in the article/Supplementary Material, further inquiries can be directed to the corresponding author/s.

## Ethics Statement

The animal study was reviewed and approved by the Animal Experiments Committee of Zhaoqing Dahuanong Biopharmaceutical Co., Ltd.

## Author Contributions

RC and DL designed and organized this study. XT did all the experimental work. CX, RC, DL, and XT analyzed the data and wrote the manuscript. All authors contributed to the article and approved the submitted version.

## Funding

This work was partially supported by the Provincial Scientific Research Institutions Key Areas R&D Plans (grant number 2021B0707010009), the National Natural Science Foundation of China (grants 31972660, 31900135, and 32170152), and the Zhaoqing Xijiang Innovative Team Foundation of China (grant number P20211154-0202).

## Conflict of Interest

The authors declare that the research was conducted in the absence of any commercial or financial relationships that could be construed as a potential conflict of interest.

## Publisher's Note

All claims expressed in this article are solely those of the authors and do not necessarily represent those of their affiliated organizations, or those of the publisher, the editors and the reviewers. Any product that may be evaluated in this article, or claim that may be made by its manufacturer, is not guaranteed or endorsed by the publisher.
